# The effects of high frequency subthalamic stimulation on balance performance and fear of falling in patients with Parkinson's disease

**DOI:** 10.1186/1743-0003-6-13

**Published:** 2009-04-30

**Authors:** Maria H Nilsson, Per-Anders Fransson, Gun-Britt Jarnlo, Måns Magnusson, Stig Rehncrona

**Affiliations:** 1 Department of Health Sciences, Division of Physiotherapy, Lund University, Lund, Sweden; 2 Department of Neurosurgery, Clinical Sciences, Lund. Lund University Hospital, Lund, Sweden; 3 Department of Otorhinolaryngology Head and Neck surgery, Clinical Sciences, Lund, Sweden

## Abstract

**Background:**

Balance impairment is one of the most distressing symptoms in Parkinson's disease (PD) even with pharmacological treatment (levodopa). A complementary treatment is high frequency stimulation in the subthalamic nucleus (STN). Whether STN stimulation improves postural control is under debate. The aim of this study was to explore the effects of STN stimulation alone on balance performance as assessed with clinical performance tests, subjective ratings of fear of falling and posturography.

**Methods:**

Ten patients (median age 66, range 59–69 years) with bilateral STN stimulation for a minimum of one year, had their anti-PD medications withdrawn overnight. Assessments were done both with the STN stimulation turned OFF and ON (start randomized). In both test conditions, the following were assessed: motor symptoms (descriptive purposes), clinical performance tests, fear of falling ratings, and posturography with and without vibratory proprioceptive disturbance.

**Results:**

STN stimulation alone significantly (p = 0.002) increased the scores of the Berg balance scale, and the median increase was 6 points. The results of all timed performance tests, except for sharpened Romberg, were significantly (p ≤ 0.016) improved. The patients rated their fear of falling as less severe, and the total score of the Falls-Efficacy Scale(S) increased (p = 0.002) in median with 54 points. All patients completed posturography when the STN stimulation was turned ON, but three patients were unable to do so when it was turned OFF. The seven patients with complete data showed no statistical significant difference (p values ≥ 0.109) in torque variance values when comparing the two test situations. This applied both during quiet stance and during the periods with vibratory stimulation, and it was irrespective of visual input and sway direction.

**Conclusion:**

In this sample, STN stimulation alone significantly improved the results of the clinical performance tests that mimic activities in daily living. This improvement was further supported by the patients' ratings of fear of falling, which were less severe with the STN stimulation turned ON. Posturography could not be performed by three out of the ten patients when the stimulation was turned OFF. The posturography results of the seven patients with complete data showed no significant differences due to STN stimulation.

## Background

Postural instability is one of the cardinal symptoms of Parkinson's disease (PD), and persons with PD run an increased risk of falling [1,2]. Most falls occur during functional activities, e.g. walking and turning [3], and it is common to experience near falls and a fear of falling [2,4,5]. Contributing factors to falls are numerous and affect both voluntary and reflexive movements in persons with PD. For instance, persons with PD have mobility difficulties, postural inflexibility, axial stiffness and deficits in central proprioceptive integration [6]. Balance capacity is a prerequisite for most of our daily tasks, and balance impairment has been shown to be one of the most distressing symptoms for patients with PD [7]. The balance impairment remains a limitation despite the use of pharmacological treatment [8] and levodopa has been shown to increase postural sway [9].

High frequency deep brain stimulation (DBS) in the subthalamic nucleus (STN) was introduced as a complement to pharmacological treatment for patients with severe PD. STN stimulation provides a more constant therapy throughout the day, and has been shown to reduce motor symptoms, motor fluctuations and decrease PD-medication requirements [10,11]. Whether STN stimulation can improve postural control is under debate [12]. The effect of STN stimulation alone can be studied after overnight withdrawal of anti-PD medication and by turning the stimulation OFF and ON respectively. STN stimulation alone has been shown to improve the results of the Berg Balance Scale (BBS) [13] and the postural stability test (item 30) of the Unified Parkinson's Disease Rating Scale (UPDRS) [10,11,14]. Item 30 (postural stability) of the UPDRS is the most commonly used clinical test for patients with PD that includes an external perturbation. The instructions and standardization of this item has been criticized in several studies, which is highlighted in a review article by Grimbergen et al.[6]. In comparison, posturography tests have the advantages of allowing a standardized and reproducible procedure of using external balance perturbations and a quantification of the postural responses.

Posturographic studies have shown that STN stimulation improves postural control, although Maurer et al. found that it hardly affected the patients' deficits in response to destabilizing visual tilts [9,15-17]. In some cases, assessment of quiet stance on a firm surface lacks the sensitivity to distinguish healthy subjects from patients with balance disorders [18]. A method commonly used to increase the sensitivity to detect balance deficits with posturography is to study the stability while postural control is challenged by balance perturbation through the somatosensory system using vibration of skeletal muscles or tendons [19]. Vibration applied to a muscle increases the afferent signals from the muscle spindles and creates a proprioceptive illusion that the vibrated muscle is being stretched [20]. The tonic stretch reflexes consequently induced are intended to return the vibrated muscle to its perceived original length [21]. Vibration of the neck or calf muscles often induces body movements primarily in an anterior-posterior direction [22]. One advantage with vibratory stimulation compared with balance perturbations methods that use physical movements, e.g., translation or inclination of the supporting surface, is that the stimulus effect is isolated to a single sensory input, i.e., the proprioception. Another advantage is that a vibratory stimulation can be controlled to produce a well-defined stimulation over time with a broad effective frequency spectrum. In none of the previous posturographic studies that investigated the effect of STN stimulation [9,15-17] was vibratory stimulation used on the calf muscles [23,24]. Persons with PD fall during activities [3] when balance is challenged by self generated perturbations and not when challenged by external perturbations. Accordingly, it is important to incorporate assessments that mimic activities of importance in daily living. When assessing balance impairment in persons with PD, it has been recommended to use an extended functional assessment of balance performance and a subjective assessment of fear of falling [5,25-27].

To our knowledge, no previous study has investigated the effect of STN stimulation alone by combining all of the above aspects that may underpin balance impairment in persons with PD. That is, combining an extended battery of clinical performance tests, subjective ratings of fear of falling and laboratory assessments that investigate reflexive movements. The aim of the present study was to explore the effects of STN stimulation alone on balance performance as assessed with clinical performance tests, subjective ratings of fear of falling and posturography.

## Materials and methods

### Patients

Ten patients (median age 66, range 59–69 years) with PD were included in the study (Table 1). Inclusion criteria were patients with PD between 59–69 years old who were treated with bilateral STN stimulation for at least one year in order to ensure a stable DBS treatment. All patients were recruited from the Department of Neurosurgery, Lund University Hospital, and the neurosurgical procedure has been described elsewhere [13].

**Table 1:** Patients' characteristics (n = 10, 9 men and 1 woman)

	**Median (range)**
Age (years)	66 (59–69)
Height (m)	1.76 (1.66–1.90)
Weight (kg)	77 (60–95)
Duration of disease (years)	18 (10–22)
Duration of DBS treatment (months)	37 (15–70)
DBS parameter settings^1^	
Right (amplitude: V, pulsewidth: μs, frequency; Hz)	3.3 (2.5–4.3), 60 (60–90), 145 (100–185)
Left (amplitude: V, pulsewidth: μs, frequency; Hz)	3.4 (2.2–4.3), 60 (60–90), 130 (100–185)
Localization of the contacts with negative polarity	
Right	11.7 (10.4–13.1) mm lateral to the midsagittal plane through the intercommissural line (IC), 3.4 (3.0–4.0)mm posterior to the midpoint of IC and 2.1 (1.0–5.6)mm inferior to IC.
Left	11.4 (9.6–13.0) mm lateral, 3.5 (3.3–5.2) mm posterior, and 2.6 (1.2–4.2) mm inferior to IC. The median length of IC was 24.8 (23.5–25.6) mm
L-dopa equivalents (mg/day)^2^	416 (242–989)
Physical Activity Scale for the Elderly (PASE)^3^	112 (75–187)
UPDRS part III^4 ^total score	DBS OFF: 41.0 (35.0–83.5), DBS ON: 21.5 (11.0–30.5)
	**n**
Occupation	10 retired (6 due to age, 4 due to disease)
Comorbidity	2 (1 patient had lumbar degenerative changes, 1 had hypertonia and a previous heart infarct)
Postural hypotension^5^	None
Prior surgery related to Parkinson's disease^6^	2 (1 pallidotomy, 1 thalamotomy + earlier DBS surgery)
Pain symptoms	6 (2 hip pain, 2 back pain, 2 shoulder pain)
Assistive device (walking indoors)	None
Assistive device (walking outdoors)	1 (cane)
Falls within the past 6 months^7^	7 patients reported falls (range 1–15 falls), whereof 5 patients reported at least 2 falls
	3 patients reported no falls: 1 experienced near falls every week, and 2 every month (whereof one of the two had fractured twice due to falls, but the last incidence was a year before the study)

DBS: Deep Brain Stimulation; DBS OFF: stimulation turned off; DBS ON: stimulation turned on.

^1 ^Polarity: Eight patients had monopolar stimulation and two patients bipolar, which applied both to the left and right hemisphere. ^2 ^L-dopa equivalents are calculated as in one of our previous studies [13]. ^3 ^Higher scores on the PASE [28,29] reflect higher level of physical activity. The mean PASE score norm for healthy men (age 65–69) is 144 and the mean PASE score in this study was 123. ^4 ^UPDRS part III: Unified Parkinson's Disease Rating Scale, motor examination [14]. Each item is graded 0–4, and the maximum total score is 108 points (higher scores reflect more severe motor symptoms). ^5 ^Clinical symptoms, combined with a systolic blood pressure drop by at least 20 mmHg (from lying to standing). ^6 ^The patient with prior DBS surgery had exchanged the target from (bilateral) Globus Pallidus internus to STN. ^7 ^A fall was defined as an unexpected event in which the patients came to rest on the ground, floor or other lower level. A near fall was defined as: a fall initiated but arrested by support from a wall, railing, other person etc. [3].

Twenty-five patients fulfilled (22 men, three women) the inclusion criteria, but 14 patients were excluded due to the following exclusion criteria: concomitant diseases interfering with balance testing, an inability to cooperate or an inability to stand for two minutes without support. One patient declined participation.

The ten included patients had all been followed up within six months before the study start. A routine clinical neurological examination was then performed, and if needed the DBS and medication was adjusted to optimize the treatment effect. The local ethical committee, Lund University, approved the study and all patients gave their written informed consent.

### Procedure & assessments

The patients were assessed as inpatients. Demographic data were collected at admission. The patients were asked to estimate their fall incidence during the past six months, and if they had experienced any near falls (for definitions see Table 1). The Physical Activity Scale for the Elderly was administered (Table 1), and this questionnaire has been tested for validity and reliability in the elderly [28,29]. As a pre-assessment trial, the physiotherapist (PT) assessed the patients when they felt at their best with their regular treatment, i.e. both anti-PD medication and STN stimulation. One leg stance and sharpened Romberg were then performed bilaterally in order to select the preferred leg (with best results) for the tests on the following day.

In order to investigate the effect of STN stimulation alone, all anti-PD medications were withdrawn overnight (from 10 pm). On the following morning, orthostatic blood pressure was measured before an independent person programmed the DBS to either ON or OFF. In order to avoid any systematic differences and bias, there was a randomization performed before the start of the study. Five patients were randomized (sealed envelopes) to begin the assessments with the STN stimulation turned ON (Deep Brain Stimulation turned on, DBS ON), and five patients with the stimulation turned OFF (DBS OFF). The PT was blinded to the randomization order.

Thirty minutes after programming the DBS, the assessments were performed in the following order: motor symptoms, clinical performance tests, subjective ratings of fear of falling and posturography. The order of the tests was chosen out of practical reasons. Short breaks were allowed between the individual tests if needed. One test session took at its most two hours, and the DBS was then reprogrammed by an independent person. During the following 30 minutes the subject had a break and a light meal (fruit, sandwich and mineral water). The second test session was then repeated with the individual tests in the same order.

In order to describe the severity of motor symptoms, the UPDRS part III (motor examination) [14] was assessed by a nurse or a neurologist (Table 1). Each patient was always assessed by the same examiner. Both examiners were experienced in using the UPDRS part III and they were trained together. The maximum total score of UPDRS part III is 108 points, and higher scores reflect more severe motor symptoms.

#### Clinical performance tests

The PT (MHN) assessed the patient with clinical performance tests, and the same PT assessed all patients in all test situations. The tests were out of practical reasons performed in the following order: the 10 m walk test, the BBS, Chair-stand Test, Timed Up & Go (TUG), One leg stance and Sharpened Romberg. One leg stance and Sharpened Romberg were performed last since the patient then needed to be barefoot.

The BBS includes 14 items (graded 0–4), and the maximum score is 56 points where higher scores denote better balance performance [30-32]. Both the BBS and the timed clinical performance tests have previously been tested for validity and reliability in the elderly and in patients with PD [30,31,33-36]. Detailed descriptions and standardizations of the timed performance tests are given in Table 2. The values obtained at the pre-assessment trial are given in Table 3.

**Table 2:** Standardizations of timed clinical performance tests

**Test**	**Procedure**
10 m walk test[36]	The subject is standing still and then walks at a comfortable (preferred) speed straight forward. The subject's regular footwear is used. Timing commence after the commando "Go'', and stops when the subjects passes the mark for ten meters. One trial is performed.

Chair-stand test [33]	The time required to stand up (erect) from a chair and to sit down five times consecutively as fast as possible is registered. The subject is sitting in an armchair (seat height of 46 cm) with the back against the chair, and with arms folded across the chest. The subject's regular footwear is worn. The test begins with the commando "Start now''. Timing commence when the subject's back is leaving the back of the chair, and stops when the subject's buttock reaches the seat for the fifth time. One trial is performed.

Timed Up & Go [34,36]	The subject is sitting in an armchair (seat height of 46 cm) with the back against the chair and arms resting on the chair's arms. The instruction "Go'' initiates the subject to stand up and walk at a comfortable (preferred) pace to a line 3 meters away, where both feet should pass the line before the subject turns around and walks back to sit down again. Timing commence when the subject's back is leaving the back of the chair, and stops when the buttock reaches the seat of the chair. The subject's regular footwear is used and customary walking aid, but no physical assistance is given. Two trials are performed. Best value is registered.

One leg stance [35]	The subject is standing barefoot on preferred foot, and freely in the room (at least 2 meters from any wall) with arms hanging. The instruction is to flex the hip and knee just enough so that the foot leaves the floor, without touching the other leg. The commando "Start'' is given, and timing commence when the foot clears the ground. Timing stops when the supportive foot moves, the lifted leg/foot touches the other leg or the ground, or the upper time limit of 60 seconds is achieved. Two trials are performed. Best value is registered.

Sharpened Romberg [35]	The subject is standing barefoot with the feet placed on a line in front of each other, toes touching the heel of the other foot. The test is performed on preferred foot (placed as the rearmost) with straight knees and arms hanging. Timing commence after achieving the position, with or without outside assistance. After conducting two trials, another two trials are conducted with eyes closed. Timing is interrupted when the subject moves either foot, opens their eyes or if the upper time limit (60 seconds) is accomplished. Best value is registered.

Time is registered in seconds, and gaitspeed is calculated as meters per second (m/s). A digital stopwatch is used. In the study by Smithson et al. One leg stance (OLS) was performed with the opposite knee flexed at 45 degrees, and the upper time limit was 30 seconds for OLS and Sharpened Romberg [35]. The standardizations for timing of the 10 m walktest, the Chair-stand test and Timed Up & Go, are in the present study described in more detail [33,34,36].

#### Ratings of fear of falling

The Falls-Efficacy Scale measures self-perceived fear of falling during ten common activities [37]. The Swedish version, FES (S), is extended with three additional activities: getting in and out of bed, grooming and toileting [38]. The FES(S) was originally tested in stroke patients, but the 13 item version has been used when investigating patients with PD [39]. Falls efficacy for the 13 activities is rated on a 10-point visual analogue scale ranging from 0: not confident at all, to 10: completely confident (Additional file 1). The maximum score is 130 points. The PT read the questions aloud and recorded the answers, and the patients performed their ratings with reference to their present status.

#### Posturography

Posturography was performed in a balance laboratory (P-A.F, JL) and conducted both with eyes open and with eyes closed. The starting order was randomized so that the patients were allocated equally. The patients were allowed to step down from the force platform and relax for three minutes in-between the tests (eyes open, eyes closed). The same test order was maintained during the DBS OFF and ON measurements.

In every test situation, spontaneous sway was recorded for 30 seconds (quiet stance) before each subject was exposed to vibratory stimulation on the calf muscles during 205 seconds. The participants were instructed to stand erect, but not at attention, on the force platform with their arms crossed over the chest. The feet were kept at an angle of about 30 degrees open to the front and with the heels approximately 3 cm apart. With eyes open, the participants focused on a mark on the wall (distance 1.5 m).

Vibratory stimulation was applied simultaneously to the middle of the gastrocnemius muscles bilaterally. The vibrators had a vibratory amplitude of 1.0 mm and a vibration frequency of 85 Hz. The vibration was produced using a revolving DC-motor (Escap, Geneva, Switzerland) equipped with a 3.5 g weight attachment contained within a cylindrical plastic coating with dimensions of 6 cm in length and 1 cm in diameter. The vibrators were secured in place by elastic straps around the legs. The vibratory stimulations were applied according to a pseudorandom binary sequence schedule [40]. This schedule defined the periodicity of stimulation shifts where each shift had random time duration from 0.8 seconds up to 6.4 seconds, which yielded an effective bandwidth of the test stimulus in the region of 0.1–2.5 Hz.

The force platform (developed in cooperation with the department of Solid Mechanics, Institute of Technology, Lund University) recorded the forces actuated by the feet with six degrees of freedom and with an accuracy of 0.5 newton. Data were sampled at 50 Hz by a computer equipped with an analogue digital converter. A customized program controlled the vibratory stimulation as well as sampling of force platform data.

### Calculations and Statistical analysis

Group results are given as medians with the first and third quartiles (q_1_–q_3_), and/or ranges.

In order to investigate the effect of STN stimulation alone, comparisons were made between DBS OFF (Deep Brain Stimulation turned off) and DBS ON after an overnight withdrawal of anti-PD medication. The Wilcoxon matched-pairs signed-ranks test was used for all comparisons. Two-tailed p-values < 0.05 were considered statistically significant, and p-values were presented exactly except when above 0.3 and below 0.001.

During posturography, the anteroposterior and lateral body movements were recorded by the force platform and quantified by analyzing the variance of the torque induced towards the ground by the body movements. Values were obtained for five periods: quiet stance (0–30 s) and from four 50-second periods during calf vibration (period 1: 30–80 s; period 2: 80–130 s; period 3: 130–180 s; period 4: 180–230 s). The torque variance values were normalized relative each subject's squared height and squared mass, compensating the torque values for individual variations in body constitution. For the posturography results, comparisons between DBS OFF and ON were done for each of the five time periods. This was conducted for anteroposterior and lateral sway, respectively, and both with eyes open and closed.

SPSS 12.0 (Chicago, Illinois, USA) was used for the calculations.

## Results

### Clinical performance tests and fear of falling

STN stimulation alone significantly (p = 0.002) increased the total score of the Berg balance scale, and the median improvement was 6 points (Table 3). Furthermore, the results of all timed clinical performance tests, except for sharpened Romberg, were significantly (p ≤ 0.016) improved with DBS ON (Table 3). All patients could perform the clinical performance tests with DBS ON. Missing data existed only with DBS OFF due to an inability to perform few of the separate tests (Timed Up & Go: one patient, Chair stand test: two patients). The patients rated their fear of falling as less severe with DBS ON as compared to DBS OFF, and the total score of FES(S) increased (p = 0.002) in median with 54 points (Table 3).

**Table 3:** Results on timed clinical performance tests, the Berg balance scale, and FES (S), n = 10

	**Admission day** **With anti-PD medication**		**Without anti-PD medication**	**Comparison between DBS OFF and ON**	
	DBS ON	DBS OFF	DBS ON	Median difference	p-value
	Md (q_1_–q_3_) range	Md (q_1_–q_3_) range	Md (q_1_–q_3_) range	Md (q_1_–q_3_) range	
Timed tests					
10 m walk test, gaitspeed (m/s)	1.3 (1.1–1.4) 1.0–1.7	0.91 (0.74–1.3) 0.38–1.4	1.3 (1.1–1.4) 0.71–1.4	0.30 (0.00–0.49) -0.06–0.73	0.016 (2 ties)
Chair-stand test (s)	15.0 (12.5–17.5) 10.0–23.0	18.5 (16.3–22.5)^1 ^ 13.0–24.0	14.5 (12.0–18.8)^1 ^ 12.0–21.0	3.5 (3.0–5.0) 1.0–6.0	0.008^1^
Timed Up & Go (s)	10.0 (8.5–11.0) 7.0–12.0	11.0 (11.0–18.5)^2^ 8.0–29.0	9.0 (8.5–11.0)^2 ^ 7.0–17.0	3.0 (1.5–8.5) 0.00–12.0	0.008^2 ^ (1 tie)
One leg stance (s)	20.5 (7.3–56.3) 2.0–60.0	11.0 (7.8–15.0) 3.0–29.0	25.5 (14.8–36.5) 3.0–47.0	11.5 (6.3–17.5) -7.0–39.0	0.006
Sharpened Romberg (s) (eyes open)	32.5 (17.0–60.0) 5.0–60.0	14.0 (6.5–27.8) 2.0–60.0	26.5 (17.0–55.5) 5.0–60.0	11.5 (-3.3–32.0) -9.0–55.0	0.051
Sharpened Romberg (s) (eyes closed)	8.0 (5.8–19.3) 2.0–32.0	4.5 (2.0–12.5) 1.0–25.0	3.0 (3.0–8.5) 2.0–14.0	1.0 (-6.3–2.3) -22.0–4.0	> 0.3

BBS	49.5 (43.8–51.5) 40.0–54.0	42.0 (34.5–48.0) 27.0–50.0	50.0 (46.8–52.0) 41.0–52.0	6.0 (2.8–12.5) 1.0–21.0	0.002

FES (S) Total score		52.5 (31.5–65.0) 3.0–95.0	111.0 (84.5–127.3) 52.0–130.0	53.5 (30.3–75.5) 21.0–100.0	0.002

Values are given as median (Md), first and third quartiles (q1–q3) and range. P values: Deep Brain Stimulation (DBS) ON as compared with DBS turned off (DBS OFF) when tested without anti-PD medication (withdrawal of all anti-parkinsonian drugs for 10–12 hours). Results are rounded as one decimal or two meaningful digits (maximum of two decimals are given).

m/s = meters per second, s = seconds.

BBS: The Berg Balance Scale, best possible score is 56 points [30-32].

FES (S): Falls -Efficacy Scale, Swedish version. Best possible total score is 130 points [38].

^1 ^(n = 8). Two patients were unable to perform the Chair-stand Test with DBS OFF, but managed with DBS ON (21 s, and 17 s).

^2 ^(n = 9). One patient was unable to perform TUG unaided with DBS OFF, but managed with DBS ON (11 s). With DBS ON, four patients had decreased results on some of the timed performance tests. All of these four patients maintained the position of sharpened Romberg with eyes closed for a shorter time period (range 3–22 s), and three out of the four did so also when tested with eyes open (range 2–9 s). One of the patients did in addition also have a slower gait speed (0.06 m/s), whereas another patient performed the One leg stance for a shorter time period (7 s). Three out these four patients had been randomized to start the assessments with DBS OFF.

One leg stance and sharpened Romberg (SR) had an upper time limit of 60 seconds. When tested without anti-PD medication, the upper time limit was reached only on the SR with eyes open (EO): one patient with DBS OFF and two patients with DBS ON. With anti-PD medication (on admission day), the upper time limit was reached by four patients while performing SR (EO) and by two patients when performing one leg stance. None of the patients had any episodes of freezing during the timed performance tests.

### Posturography

With DBS OFF, three patients were unable to perform posturography without support and they were therefore excluded from the statistical evaluation and result presentation. These three patients had the most severe resting tremor according to Item 20, UPDRS part III. With DBS OFF, their score ranged from 8 to 10 points, whereas the rest of the patients ranged between 0–3 points. Two out of the three patients had been randomized to start the assessments with DBS ON.

The remaining seven patients showed no statistical significant differences (p values ≥ 0.109) in torque variance values between DBS OFF and DBS ON (Table 4). This applied both during quiet stance and during the different periods with vibratory stimulation, and it was irrespective of visual input and sway direction (Table 4).

**Table 4:** Posturographic results: torque variance values [Nm/(kg*m)]^2^, n = 7

	**Eyes Open**			**Eyes Closed**		
Sagittal sway	DBS OFF	DBS ON	p-value	DBS OFF	DBS ON	p-value

Quiet stance	0.73 0.54–0.80	0.74 0.28–0.87	> 0.3	0.68 0.56–1.5	1.0 0.45–1.1	> 0.3
Period 1	5.9 2.8–9.1	3.8 1.9–7.3	0.219	8.6 6.8–10.9	12.0 5.5–13.0	> 0.3
Period 2	3.5 2.6–4.4	3.5 2.5–4.4	> 0.3	6.7 5.3–8.6	6.7 4.9–11.3	> 0.3
Period 3	3.3 2.7–5.8	4.5 3.2–6.4	> 0.3	10.9 6.3–12.5	8.8 6.5–13.7	> 0.3
Period 4	2.7 2.4–4.3	3.4 2.9–6.2	> 0.3	6.8 4.9–8.9	7.1 4.0–9.1	> 0.3

Lateral sway	DBS OFF	DBS ON	p-value	DBS OFF	DBS ON	p-value

Quiet stance	0.10 0.09–1.0	0.17 0.04–0.44	> 0.3	0.36 0.12–0.72	0.20 0.07–0.39	0.109
Period 1	1.1 0.64–5.2	0.66 0.48–1.4	0.156	1.1 0.87–2.7	1.1 0.65–1.5	> 0.3
Period 2	0.46 0.43–1.2	0.70 0.30–0.86	> 0.3	1.2 0.54–1.5	0.76 0.67–1.1	> 0.3
Period 3	0.64 0.26–0.96	0.52 0.26–0.75	> 0.3	0.99 0.57–1.6	0.93 0.77–1.2	> 0.3
Period 4	0.49 0.30–0.76	0.70 0.25–0.94	> 0.3	1.2 0.37–2.2	0.73 0.63–1.2	> 0.3

Torque variance values [Nm/(Kg*m)]^2 ^are given as medians and first and third quartiles. Results are rounded as one decimal or two meaningful digits (maximum of two decimals are given).

Parkinson's disease: PD.

P values: Deep Brain Stimulation (DBS) ON as compared with DBS turned off (DBS OFF) when tested without anti-PD medication (withdrawal of all anti-PD drugs for 10–12 hours).

Three patients were unable to perform the posturography unaided with DBS OFF and were therefore excluded from the statistical evaluation and result presentation.

Quiet stance: Spontaneous sway was recorded for 30 seconds.

Period 1–4: Vibratory stimulation on the calf muscles. Each period lasted for 50 seconds.

The vibratory stimulation increased the anteroposterior and lateral torque variance values significantly (p ≤ 0.047) from quiet stance to period 1 in all test conditions (DBS OFF and ON, eyes open and closed).

## Discussion

The main finding of this study is that STN stimulation alone improves clinical performance tests that mimic activities of daily living, and that it decreases the patients' fear of falling. These findings were however not supported by the posturography results although this could be a consequence of the small sample size.

### Clinical performance tests and fear of falling

The advantages and benefits of using clinical tests are that they are easy to administer, inexpensive, need no sophisticated equipment and can reflect daily activities. Using performance tests is a necessity in the clinical practice and for optimizing the effect of STN stimulation. Falls in PD tend to occur during daily activities such as walking and turning [3], and in this study all included patients did report falls or near falls. In the present study, the majority of the included clinical performance tests mimic activities in daily life. The Berg balance scale (BBS) assesses functional balance performance, and STN stimulation alone improved the BBS-results in median with six points. This is in concordance with the results of our previously published prospective study [13].

Persons with PD who have difficulties standing up from a chair have been shown to have an increased risk of falling [41]. In the present study, STN stimulation alone enabled the patients to perform both the Chair-stand test and the TUG faster. In a study by Vrancken et al., STN stimulation in combination with levodopa increased trunk flexion velocity while rising during the Get Up & Go test [42]. Previous studies have shown that STN stimulation improves gait speed and this mainly due to an increased step length [10,11,43]. Lim et al. investigated the smallest detectable difference (SDD) for the 10 m walk test (SDD 0.19 m/s) and for the TUG (SDD 1.63 s) [36]. In the present study, STN stimulation increased gait speed in median with 0.30 m/s and TUG with 3 seconds. In comparison to walking straight forward, TUG demands more complex sequences of movements. Patients with PD often have difficulties in sequential movements such as rising and turning around. The latter probably explains why one patient was unable to perform TUG with DBS OFF but managed the 10 m walk test.

The Sharpened Romberg test (eyes open and closed) was in fact the only clinical performance test that did not show any statistical significant difference between DBS OFF and ON. One reason for this could be the small sample size, and one might argue that the results with eyes open were close to significant (p = 0.051). The ceiling effect of Sharpened Romberg (eyes open) may however indicate that this test is not sensitive enough when assessing balance performance in people with PD.

The effects of STN stimulation seems more obvious when using assessments that incorporate more dynamic balance control in comparison to tests that mimic quiet stance.

Fear of falling is common among persons with PD [2,5], and it has a negative impact both on activity and participation. To our knowledge, assessments of fear of falling have not previously been included in studies when investigating the effect of STN stimulation. In the present study, the patients rated their fear of falling as less severe with the STN stimulation turned ON which supports the improvements found in the majority of the clinical performance tests.

### Posturography

The results obtained from posturography may give an ambiguous answer regarding the importance of STN stimulation in handling external balance perturbation evoked by vibratory proprioceptive stimulation, i.e. on automatic control. On one hand, three patients required external support during the posturography with DBS OFF, while all ten patients managed the posturography trials with DBS ON. That is, three out of the ten patients could not control stance when perturbed without STN stimulation, but could do so when the stimulation was turned on.

On the other hand, the posturography results of the seven patients with complete data, showed no statistical significant difference when comparing DBS ON with DBS OFF.

Although the results should be interpreted cautiously due to the small sample size, the results might suggest that STN stimulation does not markedly change peripherally triggered postural reactions if patients already with DBS OFF could withstand the perturbing stimuli.

Earlier studies have shown that patients with PD are particularly unstable when perturbed backwards [44,45], and vibratory stimulation on the calf muscles gives the perception of being pulled backward [22]. None of the previous posturographic studies that investigated the effect of STN stimulation did use vibratory stimulation as an external perturbation, which makes comparisons difficult [9,15-17,46]. It is often complex to compare posturographic studies since different perturbations often have been used and the results are presented in diversified ways.

Thus, the posturography results in the present study did not support the improvement seen in the clinical performance tests. This may indicate that STN stimulation is less effective on automatic postural responses compared to the effect on balance control required during activities. Alternatively, it may be explained by the fact that posturography could only be made on patients that could withstand the perturbing stimulus. In fact three patients could do so with DBS ON, but not when the DBS was turned OFF. This is similar to a previous observation in stroke patients, where the number of patients that could withstand calf vibration doubled after therapeutic sensory stimulation with acupuncture [47]. Neither in this study was there any difference in sway parameters among those that could cope with the perturbations.

The aim of the present study was to investigate the effect of STN stimulation alone. In daily life, the patients are however treated with STN stimulation in combination with reduced dosage of anti-PD medication. Prospective studies of how the combined treatment affects balance performance, fear of falling and fall incidence are therefore warranted.

## Conclusion

In this sample, STN stimulation alone significantly improved the results of the clinical performance tests that mimic activities in daily living. This improvement was further supported by the patients' ratings of fear of falling, which was less severe with the STN stimulation turned ON. Posturography could not be performed by three out of the ten patients when the stimulation was turned OFF. The posturography results of the seven patients with complete data showed no significant differences due to STN stimulation.

## Competing interests

The authors declare that they have no competing interests.

## Authors' contributions

MN participated in the design of the study, recruited patients, managed acquisition of data, performed data analysis and drafted the manuscript.

PAF participated in collecting posturographic data, assisted in data analysis and in drafting the manuscript.

GBJ participated in the design of the study and helped draft the manuscript.

SR and MM participated in the project organization, design, supervised the project and helped draft the manuscript.

All authors read and approved the final manuscript.

## Supplementary Material

Additional file 1 **Appendix A**. Appendix A describes the Falls-Efficacy Scale, Swedish version – FES(S) Click here for file
