# Supplementary material for: The effects of high frequency subthalamic stimulation on balance performance and fear of falling in patients with Parkinson's disease
Source: J Neuroeng Rehabil. 2009 Apr 30;6:13. doi: 10.1186/1743-0003-6-13 (PMC2683854; doi:10.1186/1743-0003-6-13)
Supplement: Additional file 1 — Appendix A. Appendix A describes the Falls-Efficacy Scale, Swedish version – FES(S) [file 1743-0003-6-13-S1.pdf]

Appendix A

Falls-Efficacy Scale, Swedish version – FES(S)												
Question	How confident/sure are you that you can….without falling?											
Item	Activity	Visual analogue scale										
		Not Confident at all					Fairly Confident					Completely Confident
		0	1	2	3	4	5	6	7	8	9	10
1	Get in and out of bed											
2	Get on and off the toilet											
3	Personal grooming											
4	Get in and out of chair											
5	Get dressed and undressed											
6	Take a bath or a shower											
7	Go up and down stairs											
8	Walk around neighborhood											
9	Reach into cupboards/closets											
10	Housecleaning											
11	Prepare simple meals											
12	Answer the telephone											
13	Simple shopping											

Tinetti et al. developed the original FES which included items 4-13. The Swedish version is developed by Hellström et al. who added three additional items (items 1-3).
The question (How confident/sure are you that you can….without falling?) is read for each of the 13 activities. Each activity is rated on a visual analogue scale (graded from 0: not confident at all to 10: completely confident).

A physical therapist (PT) asked the patients all 13 questions and recorded the answers. The PT showed the visual analogue scale for the patients, who were instructed to rate according to their present status.

Tinetti ME, Richman D, Powell L: Falls efficacy as a measure of fear of falling.
J Gerontol 1990, 45:P239-243.
Hellstrom K, Lindmark B: Fear of falling in patients with stroke: a reliability study.
Clin Rehabil 1999, 13:509-517.
